# Care of Transgender Patients: A General Practice Quality Improvement Approach

**DOI:** 10.3390/healthcare10010121

**Published:** 2022-01-07

**Authors:** Isabel Boyd, Thomas Hackett, Susan Bewley

**Affiliations:** 1 Falmouth and Penryn Primary Care Network, C/O Penryn Surgery, Saracen Way, Penryn, Cornwall TR10 8HX, UK; 2 Penryn Surgery, Saracen Way, Penryn, Cornwall TR10 8HX, UK; thomas.hackett1@nhs.net; 3 Department of Women and Children’s Health, School of Life Course and Population Sciences, Faculty of Life Sciences & Medicine, King’s College London, London SE1 7EH, UK; susan.bewley@kcl.ac.uk

**Keywords:** gender dysphoria, transgender, general practice, primary care

## Abstract

Primary care must ensure high quality lifelong care is offered to trans and gender minority patients who are known to have poor health and adverse healthcare experiences. This quality improvement project aimed to interrogate and audit the data of trans and gender minority patients in one primary care population in England. A new data collection instrument was created examining pathways of care, assessments and interventions undertaken, monitoring, and complications. General practitioners identified a sample from the patient population and then performed an audit to examine against an established standard of care. No appropriate primary care audit standard was found. There was inconsistency between multiple UK gender identity clinics’ (GIC) individual recommended schedules of care and between specialty guidelines. Using an international, secondary care, evidence-informed guideline, it appeared that up to two-thirds of patients did not receive all recommended monitoring standards, largely due to inconsistencies between GIC and international guidance. It is imperative that an evidence-based primary care guideline is devised alongside measurable standards. Given the findings of long waits, high rates of medical complexity, and some undesired treatment outcomes (including a fifth of patients stopping hormones of whom more than half cited regret or detransition experiences), this small but population-based quality improvement approach should be replicated and expanded upon at scale.

## 1. Introduction

Transgender (trans) people experience adverse health outcomes and increased mortality [1,2]. Poor healthcare has been identified as one of many factors that lower quality of life for trans people [3]. A 2016 Lancet series examined in detail the conditions in which transgender people live and experience healthcare and provided a framework on which to make improvements to social and legal conditions as well as clinical care considerations and service delivery models [4]. Transgender people report that when they access healthcare regarding gender dysphoria, providers often lack relevant skills, and appear unsupportive or hostile to their healthcare needs [5]. They feel uncomfortable discussing their needs with new health care providers and have a fear of mistreatment [6]. These significant challenges were supported by a recent systematic review of qualitative experiences [7]. Indeed, transgender patients have called for health care providers to have greater education on transgender issues [8] and to recognize more individual, diffuse combinations of care and transitions over time [9]. There is little current primary care population-based clinical data about transgender health. It is thus timely for all doctors, especially those with first contacts or responsible for life-long healthcare delivery in primary care systems, to consider their own education, their patients’ experiences, and how to measure and improve quality.

International specialist guidelines exist for the treatment of gender dysphoria. These include the World Professional Association of Transgender Health (WPATH) guidelines [10] and the Endocrine Society guidelines [11], both of which are widely cited. An eighth Standards of Care from WPATH is in the process of being issued in early2022 [12]. Some guidance for primary care has been published by the University of California San Francisco [13] but will not necessarily be relevant to other countries with different primary care systems. The United Kingdom (UK) has a universal health care system funded out of general taxation, the National Health Service (NHS) with a small insurance and self-paying private sector. Care is commissioned based on a General Practitioner (GP) primary care model, referring to secondary care (hospital-based) specialists who share care with, or return patients for continuing care back to primary care.

In 2019 NHS England (the NHS commissioning board) published Service Specifications for Gender Identity Services for adults [14]. NHS England service specifications are written to define standards of care expected from specialist services. They are developed by specialized clinicians, commissioners, expert patients, and public health representatives. These guidelines recommend either self-referral or GP referral to Gender Identity Clinics (GICs), followed by a minimum of two assessments in the clinic where neither endocrinology nor psychology input is considered mandatory, and support should be “affirmative”. The clinics are then permitted to ask the patient’s GP to prescribe hormones and/or gonadotrophin-releasing hormone agonists (GNRHa) under a “Shared Care Agreement” where the clinic provides ongoing follow-up of the patient until they are discharged. The shared care agreements also include monitoring instructions for the GPs to follow in terms of physical checks, blood tests, and screening. NHS England states that GPs should “co-operate with the specialist Gender Identity Clinics and prescribe hormone therapy” [15]. The service specifications describe “quality indicators” for secondary care which include monitoring for the following complications only; myocardial infarction, stroke, deep vein thrombosis, pulmonary embolism, and polycythaemia [14]. NHS England does not recommend monitoring for compliance or concordance with hormone prescriptions, or for cessation of treatment or detransition.

In terms of regulation, pharmacological interventions for gender dysphoria are not licensed by the UK Medicines and Healthcare Regulatory Agency with the exception of Sustanon. The General Medical Council (GMC) (the UK’s regulatory body for doctors) advises that when prescribing unlicensed medications, doctors “must be satisfied that there is sufficient evidence or experience of using the medicine to demonstrate its safety and efficacy and must take responsibility for prescribing the medicine” [16]. Regarding prescribing under shared care agreements, the GMC advises that doctors must be satisfied that the prescription is “within the limits of competence” [17]. However, the GMC provides separate, contradictory advice regarding trans healthcare where they say it would not be acceptable to “refuse to treat the patient” [18]. These discrepancies in guidance, with their legal implications, have been challenged by the British Medical Association (the largest doctors’ trade union) and the Royal College of General Practice (RCGP) [19,20,21].

Regarding education, the results of a systematic review of interventions to enhance training of health professionals in sexual and gender minority issues of patients are awaited [22]. Equality and diversity issues have been incorporated into the GP training curriculum [23] and three e-modules on gender variance and transgender healthcare are available; two for Members of the RCGP [24] and one open access [25]. In 2021, the Royal College of Physicians launched two Certificate and Master’s training courses designed for special interests in Gender Identity Healthcare [26].

Clinical guidance also exists within two of the devolved nations of the UK; NHS Scotland [27] and NHS Wales [28]. There are also continuing medical education and best practice articles available [29,30]. However, as yet neither the National Institute for Health and Care Excellence (NICE) [31] nor the British Association of Gender Identity Specialists [32] have produced nationally agreed evidence-based assessment and treatment guidelines for gender dysphoria in the UK (in either primary or secondary care), or standards of care or quality measures for gender minority/trans patients in primary care.

In terms of shared decision aids that GPs might use, no reliable data derived from randomized controlled trials, or longitudinal cohort studies give confident estimates of the risks and benefits of various gender-affirming interventions. A widely cited “systematic review” uses no comparators [33]. Long-term patient outcomes are uncertain, partly due to high losses to follow up. The precision of cohort and linkage studies may be affected by sex and gender marker changes [34], and the issuing of new NHS numbers where gender can be changed from the sex marker assigned at birth [35]. In the UK, patients can request new NHS numbers at any point in their transition process. For confidentiality purposes, any reference to their previous gender and gender transition is redacted.

Referrals to GICs have increased in recent years [36]. In response to seeing increasing patients with gender dysphoria, one GP surgery in Southwest England responded by deciding to audit the quality of care that their patients were receiving.

## 2. Materials and Methods

### 2.1. Setting

This local quality improvement project combined a practice population census, single-stage review of health records, and audit against standards in order to examine the surgery’s prescribing and monitoring against a backdrop of patient demographics and treatment pathways.

The GP practice population was used as a sampling strategy. It is a multisite training practice situated in Southwest England, where 10¾ full-time equivalent GPs serve over 20,000 patients in a mixed rural/urban area with a nearby university. The practice had nominated a practice GP lead for transgender care, and two GPs visited the local GIC in 2019. A patient information pack had been developed, including signposting to national support agencies, information about referral processes to private and NHS GICs, and health screening advice. In 2018, the practice had been inspected by the health and social care regulator, the Care Quality Commission [37], which had rated the practice as “Good” overall. The surgery’s gender dysphoria policy requires patients carry on GIC follow-up until formal discharge for the GP to continue prescribing, bridging hormones will not be prescribed, and shared care with private clinics is accepted when the specialist demonstrates experience in gender medicine, also working in NHS gender services.

### 2.2. Searching for an Audit Standard

Searches for standards of care for gender dysphoria treatment were undertaken in PubMed, MEDLINE, Google, and guideline repositories (Guideline Central and ECRI Guidelines Trust). GIC schedules were collated from individual patient shared care agreements. Table 1 and Table 2 compare international and national prescribing and monitoring guidelines alongside local schedules from various UK NHS and private GICs that our patients attended. As shown in the Tables, there was considerable variation regarding drug dosing recommendations, target hormone levels, type of monitoring including blood tests, and recommended investigations such as pelvic ultrasound and bone density assessment. The team discussed which was the appropriate audit standard taking into consideration the following features: an evidence-based process underpinning the creation of the guidance; applicability to primary care and the UK; clarity and accessibility of instructions and transferability to all practice patients.

### 2.3. Instrument Creation

A data collection instrument was devised and piloted in order to describe our own patients, their pathways to care and perform the audit. The data collection spreadsheet was devised with quantitative answers (numerical or yes/no/missing) and free text boxes. A blank version can be downloaded at: https://www.mdpi.com/article/10.3390/healthcare10010121/s1, Table S1: Data collection spreadsheet. The practice population census and first baseline audit were performed on 16 September 2020.

### 2.4. Patients/Sampling Frame

All patients with a diagnosis of gender dysphoria were identified from the electronic patient record by performing a SystmOne report searching for Read codes transsexualism (E225), transexual (Ua1b8), gender identity disorder (X00TG), gender dysphoria (XSEDT), transgender identity (Xafge), transgender female (Y1f4e), transgender male (Y1f4f) or transgender (Y1f50). In case of coding errors, all patients taking testosterone or estradiol were also identified and excluded if prescriptions were not gender related. These Read codes may be exclusive to the UK but are described for the purpose of replicability and reproducibility in this and other countries.

### 2.5. Data Extraction

Two GPs (IB, TH) independently extracted data. All discrepancies were checked against the original record, discussed and corrected, and then anonymized. Data included: demographics (age, race, sex, gender identity, co-morbidities), age at medical presentation with gender dysphoria, time to gender interventions, description of interventions, individual monitoring plan, adherence, correspondence with GICs and complications confirmed by specialists. Gender identity included non-binary and other self-identified descriptions.

### 2.6. Strategy for Data and Statistical Analyses

Missing data were dealt with by using total denominator. Simple frequencies were calculated. No tests for statistical significance were used.

### 2.7. Ethical Considerations

This work uses data provided by patients and collected by the NHS as part of their care and support [41]. Research Ethics in the UK is governed by the Integrated Research Application System (IRAS) process; a single system for applying for the permissions and approvals for health and social care/community care research [42]. The authors worked through the joint NHS Health Research Authority (HRA) and Medical Research Council (MRC) process to determine and confirm that the project was quality improvement, not research, and that formal research ethics committee approval was not required [43].

## 3. Results

### 3.1. Choice of Audit Standard

No UK-wide nor international primary care quality standard was found that suited our purposes. In lieu of curtailing the audit cycle, secondary care standards were extracted from the Endocrine Society Guideline [11], because they were clear, had been developed after a process using systematic reviews, grading of evidence and expert consensus, and because they had been written for an international audience.

### 3.2. Description of the Population Census and Pathways

#### 3.2.1. Demographics

Sixty-eight patients (total practice list size 20,136) were identified as trans or gender minority (practice prevalence 337/100,000) at various stages along the gender dysphoria pathway. Five (7%) had not yet been referred, 15 (22%) had been referred but not yet seen in clinic and 48 (71%) had been seen in a GIC. Their mean age was 27.8 years (range 19–89, median 22). Forty-two (62%) were trans men (documented female sex at birth), 22 (32%) trans women (documented male sex at birth), and four were non-binary; three (4%) female and one (1%) male documented sex at birth. Mean age of presentation with gender dysphoria was 20 years (range 12–54, median 18). The mean age of presentation of trans men and non-binary females was 18 years. The mean age of presentation of trans women and non-binary males was 23 years. Table 3 shows past and existing psychosocial and mental health issues.

#### 3.2.2. Clinic Referrals and Pathways

Forty-eight patients had been seen in 17 different GICs (11 NHS, four UK private, one USA, one Italy). Twenty-four patients (50%) had been seen in an NHS clinic only, 15 (31%) had been seen in a private clinic only, while seven (15%) had been seen in both private and NHS clinics, and two (4%) had been seen abroad. Sixteen patients had self-referred to a GIC (all private); in only one of these cases did the GIC request information from the GP prior to recommending treatment. Twenty-five patients were waiting for an NHS appointment (including 10 whom had been seen privately). The mean waiting time was 26 months. No patients were waiting for a private appointment.

Following assessment in clinic, forty-one patients (85%) were started on hormones, two patients (four percent) were discharged without any treatment and five (10%) were awaiting further assessment in the clinic. Prescribing was initiated by the GP at the request of the GIC for 12 patients (29%), the rest were initiated by GIC barring one where a GP started hormones in the 1990s before GIC assessment. Hormones were started after a mean 2.7 appointments in clinic (3.6 NHS, 1.9 private); eight patients had hormones started after one appointment (two NHS, six private).

#### 3.2.3. Medical and Surgical Management

Eleven patients received gonadotropin-releasing hormone analogues (GnRHa). The mean age of starting GnRHa was 22 years (median 18, range 15–40). GnRHa were started in five trans women (mean age 29) and six trans men (mean age 17) and used for a mean 34 months. Patients remained on a wide range of hormone treatments at the time of the audit; ten Nebido injections, six Sustanon injections, seven Testogel, one Tostran, two Estradiol Hemihydrate, four Estradiol Valerate, one Oestrogel, one Estradot. Two patients were on a GNRHa in addition to hormone therapy. One patient had stored oocytes, none had stored sperm.

Of the 41 patients that had received hormone treatment, 20 (49%) underwent gender affirming surgery. For the trans men this consisted of: Bilateral mastectomy, total abdominal hysterectomy and bilateral salping-oophorectomy (TAH & BSO), vaginectomy and phalloplasty (one patient); bilateral mastectomy, TAH & BSO (two patients); TAH & BSO alone (one patient); and bilateral mastectomy alone (nine patients). Mean age of mastectomy was 24 years (median 20, range 17–56), and for TAH & BSO was 29 years (median 23, range 22–45). For trans women surgery consisted of: Breast augmentation plus penectomy, orchidectomy and vaginoplasty (two patients); Penectomy, orchidectomy and vaginoplasty (three patients); and Other (self orchidectomy as adolescent) (one patient). The mean age of penectomy, orchidectomy and vaginoplasty was 31 years (median 34, range 21–37).

#### 3.2.4. Undesired Treatment Outcomes (Stopping Hormones, Abnormal Blood Test Results, Side Effects and Complications)

Nine patients had stopped hormone therapy; one related to practice policy because they had not attended any GIC follow-up (the patient has restarted since the audit). Thus, eight patients had stopped hormones voluntarily (20% stopping rate; six trans men, two trans women). These patients had been on treatment for a mean of five years (range 17 months-10 years). Four transmen had comments in the records that related to a change in gender identity or detransitioning (4/41, 9.8%): “Would like to gradually detransition”; “No longer wish to live your life as a male”; “Has decided to detransition…. Feels comfortable having decided to dress and appear more feminine; “Feels it was a mistake, identifying as non-binary now”. None of these patients had undergone any gender related surgery. They had presented at a mean of 18 years of age, taken testosterone for a mean of 18 months, and currently presented as female (three) or non-binary (one). The other four patients who had stopped hormones continued to present as trans (two women, two men): one, who had experienced orchidectomy, had a record of regret (“No hormonal treatment currently, regrets gender reassignment”); one had a medical reason noted for stopping (“problems with PV bleeding despite androgen”); and two had no specific reason for stopping in their record, but it was documented that they had stopped.

In 15 cases (37%) of patients on hormones, levels had been found to be out of target range at one or more point during the course of therapy. Twelve trans men had transiently raised testosterone; one with a level of 120 nmol/L (over four-fold advised peak levels) who now has injections at the surgery instead of self administering. Three trans women had transiently raised estradiol levels above target range (highest, 1151 pmol/L). Transient polycythaemia (HCT > 0.5 L/L) was found in 9 trans men (highest haematocrit 0.57 L/L).

Five trans men (16%) had documented complications: severe acne attributed to testosterone by dermatologist (two); unwanted male pattern balding (one), vaginal bleeding (one); and vaginal atrophy requiring topical oestrogen (one). Two trans women (20%) had documented complications: severe vaginal wound infections (one resulting in sepsis requiring admission and intravenous antibiotics, the other in vaginal stenosis).

### 3.3. Audit of Monitoring against Standards

Twelve patients on treatment (38%) had all Endocrine Society recommended monitoring in the previous year: (a) all but two patients had hormone levels performed—in these two cases, their GICs had not requested annual hormone checks; (b) 14 transmen (58%) did not have all of lipids, blood pressure and weight measured in the previous 12 months (recommended by the Endocrine Society for trans men at non-specified “regular intervals” but GIC guidance on this monitoring varies—see Table 1 and Table 2, the Endocrine Society does not strongly recommend this monitoring for transwomen); (c) all 24 patients on testosterone had haematocrit checked; (d) Ten trans men post-mastectomy (100%) had not received annual sub and peri-areolar breast examinations as recommended by the Endocrine Society (no GIC recommended this).

## 4. Discussion

### 4.1. Summary of Main Findings

This first published UK quality improvement project of transgender healthcare in primary care generated useful, practice-level data. No UK-wide evidence-based treatment or monitoring guidelines for gender dysphoria in primary care were found. Existing international, national, and GIC guidelines were found to contain contradictions. This problem constrained the audit cycle but is an important finding in itself. The practice audit found that 62% of patients received at least one measure of substandard monitoring when set against the Endocrine Society guidance, largely due to conflicts with the patients’ individual GIC Schedule of Care. By contrast, the practice services for trans patients overall had been noted as “outstanding” in 2018 following an inspection by the independent UK health and social care monitor and regulator, the Care Quality Commission [37], maybe demonstrating the inadequacy of using the blunt tools of a conflicting international guideline, idiosyncratic UK GIC Schedules of Care or the CQC process.

As practice patients are being treated by multiple NHS and private clinics, provision of safe monitoring by GPs is likely to be more difficult when no national standard exists and GICs’ guidance conflict with one another. Thus, it is difficult for any primary health caregiver to know whether their care really is objectively “substandard” or not. How does a GP know which is the optimal guideline to follow? There is no mechanism for them to question whether guidance recommended by the Endocrine Society such as annual “sub and periareolar breast examinations” in transmen post-mastectomy, is evidence-based, and in patients’ interests, rather than unnecessary and invasive. A further example of conflicting guidance is demonstrated with pelvic ultrasound monitoring of transmen on testosterone (see Table 1 and Table 2). Guidance varies from no advice given, no monitoring advised, biannual ultrasound, or two-yearly ultrasound. Guidance around polycythaemia is also variable, the consequent complication of which could be a stroke or heart attack.

Aside from long waiting times, the routes to hormone therapy and surgery aligned with NHS service specifications [14]. There were many self-referrals to GICs (bypassing traditional primary care triage or gatekeeping), few assessment appointments, and high levels of GP-initiated prescribing. Self-referral was only found in the private clinics, and private clinics provided fewer assessment appointments than the NHS clinics. No international, national, or GIC guideline that we reviewed provided information regarding detransition or regret, or services for these patients.

### 4.2. Strengths and Limitations

Strengths include a population-based census cohort with iterative, piloted quality improvement approach, wide inclusion and comprehensive description of patients, and independent double data collection. Limitations include small single practice numbers from a remoter part of Britain and data collection from the electronic record only. Despite the natural emphasis on the quality of care given within the UK (and specifically, Southwest England) context, the project shows “proof of concept” that audits can (and indeed should) be performed elsewhere. Due to the specific setting, prevalence rates, including mental health problems, complications, and detransition are not generalizable to other populations of patients with Gender Dysphoria. However, they are population (rather than clinic) based and illustrate some of the complexities that may be affecting this group of patients. 

A significant proportion of patients (29%) had not yet been referred or seen in a GIC. Without contacting these patients, it was not possible to know just by reviewing their electronic records whether they were still pursuing transition or not. For this reason, the denominator chosen for the detransition rate was the number who had started hormone treatment. Similarly, the prevalence rate of gender dysphoria at this surgery may be an underestimate if patients have not presented to medical services, have undergone social transition alone, or are sourcing hormones off the internet.

### 4.3. Comparison with Existing Literature

Variability in the quality of international guidelines and the absence of primary care guidelines have been previously reported [44]. As far as we know, UK GIC monitoring guidance has not previously been compared with international guidance. Although the demographic data were slight and specific to the locale (for example, the patient prevalence at this surgery of 337/100,000 with a high number of co-existing or previous psychosocial diagnoses) it can be used to compare with other population-based data [45]. It was not the focus of this project to do formal comparisons, but such primary care data can be added to others’ prevalence estimates. A higher proportion of younger observed females at birth (transmen) presenting has been previously noted [46], along with high rates of autistic spectrum disorder [47], mental health diagnoses [48], and early trauma including sexual abuse [49].

In terms of detransition rates, one meta-analysis reports rates of less than one percent [50] but the primary studies are inherently flawed by loss to follow up. A recent UK GIC case note review found a detransition rate of 6.9% [51]. Thus, the detransition rate found in this population is novel and questions may be raised about the phenomenon of overdiagnosis, overtreatment, or iatrogenic harm as found in other medical fields.

### 4.4. Implications for Clinicians, Policy Makers, and Patients

This project makes an argument for the improvement and standardization of the care given to trans and gender minority patients. Policymakers should issue national, evidence-based assessment, treatment, and monitoring guidelines for primary and secondary care, including expected communication standards in records and between sectors. In the UK these types of guidelines are typically published by the National Institute for Health and Care Excellence [31], or by specialist societies using their accreditation system [32].

Until such guidance is available for GPs, the GMC and NHS England should review their advice, including support for GPs who may not feel it is within their competency to assess and prescribe for gender dysphoria. GPs and patients should be able to access shared decision-making aids with information about the sizes of harms and benefits, including the possibility of detransition.

### 4.5. Implications for Research

This work builds on traditional primary care-based approaches [52]. Quality improvement audits, even without completed cycles, can be hypothesis-generating and immediately replicated in other settings. Formal research could be performed, for example via the primary care academic collaborative (PACT) [53] and patient priority setting partnerships, to investigate questions about rates of gender dysphoria, the quality of care, management of co-morbidities, frequency of unwanted outcomes, and complications including detransition. NHS Child and Adolescent Mental Health Services and adult mental health services should investigate novel means of supporting young people, especially those with comorbidities or mental health issues, who presently languish on long waiting lists that may be worsening in the coronavirus pandemic, preferably using robust research methodologies. Research is needed into the current “affirmative” approach that is supported globally and by NHS England, in order to minimize potential harm to patients from complications of treatments, or their cessation. Long-term follow up, using linked national databases, could generate patient outcome data to inform primary care and secondary care practitioners about the ongoing general health needs of this important population.

## 5. Conclusions

These findings complement secondary care-generated knowledge and suggest room for improvement. Gender minority/trans people will be better served by evidence-based primary care guidance, including monitoring and communication standards. Existing guidance is contradictory. Given the findings of long waits for GIC assessment, and some unwanted outcomes and complications (including detransition), this small but hypothesis-generating primary care-focused, population-based, quality improvement approach should be replicated and expanded upon at scale.

## Figures and Tables

**Table 1:** Comparison of national, international, and patient specific prescribing and monitoring guidelines for trans men (observed females at birth) with gender dysphoria.

Hormone Dosing and Routine Long Term Monitoring (once stable/after 12–36 Months) 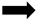	Maximum Transdermal Testosterone Dose (mg)	Minimum Nebido Injection Frequency (Weeks)	Minimum Sustanon Frequency (Weeks)	ANNUAL MONITORING																Pelvic USS monitoring	Breast Screening (unless Mastectomy)	Cervical Screening (unless Total Hysterectomy)	Bone (DEXA)	AAA Screening
				Testosterone Level	Target Testosterone Levels (nmol/L) TD = Transdermal N = Nebido S = Sustanon Tr = Trough (Day of Injecton) Pk = Peak (7 Days after Injection)	Full Blood Count	Haematocrit Levels Specific Advice (L/L)	Liver Function Tests	Lipids	Estradiol	Glucose	HBA1c	Prolactin	FSH&LH	SHBG	Urea and Electrolytes	TSH	Blood Pressure	Weight					
Guideline or Clinic 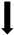																								
**International**																								
**WPATH [10]**	No specific dosing instructions given. WPATH recommends consulting Feldman and Safer [38] and Hembree et al. [39] for hormone regimes and lab monitoring protocols. Testosterone levels should be maintained “within the normal male range while avoiding supraphysiological levels”. WPATH also advises “Follow-up should include careful assessment for signs and symptoms of excessive weight gain, acne, uterine break-through bleeding, and cardiovascular impairment, as well as psychiatric symptoms in at- risk patients. Physical examinations should include measurement of blood pressure, weight, and pulse; and heart, lung, and skin exams”																	Y	Y					
**Endocrine Society [11] ^A^**	100	12		Y	11.1–34.7 ^B^	Y			Y^D^									Y^D^	Y^D^	N	Y ^E^	Y	N ^C^	
**National/Local**																								
**Australia [40]**	100	8	3	Y ^F^		Y ^F^		Y ^F^	Y	Y^F^	Y			Y ^F^		Y ^F^		Y ^G^	Y ^G^				Y ^M^	
**San Francisco [13]**	103.25	10		Y	Physiological male range	Y	If above male reference range- check testosterone level, adjust testosterone dose, short term blood donation may be the solution													N	Y	Y	Y ^H^	
**NHS Wales [28]**	80	11	2	Y	TD, N: 15–20 S: 8–12 Tr, 25–30 Pk	Y	>0.52 seek GIC advice >0.6 stop treatment & seek urgent haematology advice	Y	Y			Y						Y	Y	Biennial^P^	Y	Y		Y ^J^
**NHS Scotland [27]**	100	10	2	Y	S,N; Tr “lower 3rd of normal range” TD: “within normal male range”	Y												“CV risk assess”			Y	Y	N ^C^	
**GP CPD Red Whale [30]**	50	12	2	Y		Y		Y	Y	Y	Y		Y			Y	Y	Y			Y^Q^	Y	N ^C^	Y ^J^
**GIC guidance**																								
**London Transgender clinic (private)**		10	2	Y	TD: 15–25 N: 15–30 S: 8–12 Tr, 25–30 Pk	Y	<0.52 acceptable 0.52–0.55 increase hydration and repeat bloods before next injection or in 8 weeks 0.55–0.6 refer urgently to haematology	Y	Y		Y							Y	Y	N	Y^K^	Y^K^		Y^K^
**Laurels NHS**	80			Y	14–28	Y	>0.56 seek prompt advice from haematologist and The Laurels	Y	Y		Y							Y^F^	Y^F^	N		Y		N
**Gender GP (private)**	100	10	2	Y	9–38 Steady State 8–12 Tr	Y	>0.52 suspend testosterone and refer endocrinology	Y	Y		Y					Y				Biannual	N		N ^C^	
**Sheffield NHS**	100	10	2	Y	S, N: 8–12 Tr, 25–30 Pk TD: 15–20	Y	> 0.52 suspend treatment and refer haematology	Y	Y	Y		Y	Y			Y		Y	N ^L^	N	Y	Y	N	Y
**Tavistock NHS**	100	6	2	Y	S: 10–12 Tr, 25–30 Pk N, TD: 15–20	Y	>0.52 hydration, repeat test in 8 weeks or on day of next injection >0.55 seek GIC advice immediately >0.6 pause treatment, seek urgent GIC and haematology advice	Y	Y										Y	Biannual	Y	Y		Y^K^
**Nottingham NHS**	100	10	2	Y^R^	TD: “upper 1/2 of local ref range” S, N: “lower 1/3 of local reference range” Tr/ steady state	Y	≥0.52 routine referral to haematology ≥0.54 urgent referral to haematology	Y	Y											Biannual	Y^K^	Y^K^		
**Gendercare (private)**	100	10	2	Y	S, N: 8–12 Tr, 25–30 Pk TD: 15–20	Y		Y	Y										Y	Biannual	Y	Y		Y^K^
**Leeds NHS**	80	8	2	Y	S: “lower 3rd reference range” trough level N, TD: “middle third reference range”	Y	If ≥54% withhold treatment & discuss with specialist	Y	Y	Y		Y						Y			Y	Y		

Key: Empty cell: no specific advice given; HBA1c, Glycated Haemoglobin; FSH&LH, Follicle Stimulating Hormone and Luteinizing hormone; SHBG, Sex hormone binding globulin; TSH, Thyroid Stimulating Hormone; CV, cardiovascular; ^A^,”strong recommendations”; ^B^, “the normal male range, dependent on the assay but is typically 320–1000 ng/dL”; ^C^, Unless prolonged periods without sex hormones or additional risk factors; ^D^, “at regular intervals”; ^E^, plus conduct sub and periareolar breast examinations if mastectomy performed; ^F^, Every 6 months; ^G^, Every 3 months; ^H^, from age 65 years on (earlier if risk factors); ^J^, if patient wishes; ^K^, screening as per https://www.gov.uk/government/publications/nhs-population-screening-information-for-transgender-people/nhs-population-screening-information-for-trans-people (accessed on 1 Jan 2022); ^L^, unless considering surgery; ^M^, Annually to document recovery after being on puberty suppression as required; ^Q^, Offer screening also if any breast tissue post mastectomy; ^R^, two to three times per year long term; ^S^, and discuss technical limitations of breast screening post mastectomy, with unknown risks of breast cancer; ^T^, also offer breast screening post mastectomy.

**Table 2:** Comparison of national, international, and patient specific prescribing and monitoring guidelines for trans women (observed males at birth) with gender dysphoria.

Hormone Dosing and Routine Long Term Monitoring (once stable/after 12-36 Months) 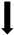	Maximum Oral Estradiol Dose (mg)	Maximum Transdermal Estradiol Dose (gel) (mg)	Maximum Transdermal Estradiol Dose (Patch)(mcg Twice Weekly)	ANNUAL MONITORING																		
				Estradiol Level	Target Estradiol Level (nmol/L)	Testosterone	Liver Function Tests	Lipids	Glucose	HBA1c	Prolactin	Full Blood Count	FSH&LH	SHBG	Urea and Electrolytes	TSH	Vitamin D and Bone Profile	Blood Pressure	Weight	Breast Screening	Bone (DEXA)	AAA Screening
Guideline or Clinic 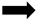																						
**International**																						
**WPATH [10]**	No specific dosing instructions given. WPATH recommends consulting Feldman and Safer [38] and Hembree et al. [39] for hormone regimes and lab monitoring protocols. Target estradiol levels “within a premenopausal female range but well below supraphysiologic levels”. Follow- up should also “include careful assessment for signs of cardiovascular impairment and venous thromboembolism through measurement of blood pressure, weight, and pulse; heart and lung exams; and examination of the extremities for peripheral edema, localized swelling, or pain”																	Y	Y			
**Endocrine Society [11] ^A^**	6		200	Y	360–735 ^B^	Y									Y ^D^					Y	Y ^E^	
**National/Local**																						
**Australia (children & adolescents) [40]**	4			Y ^G^		Y ^G^	Y ^G^	Y	Y			Y ^G^	Y ^G^		Y ^G^			Y ^H^	Y ^H^		Y ^J^	
**San Francisco [13]**	8		400	Y ^K^	physiological menstruating female range ^R^	Y ^L^									Y ^D^					Y ^M^	Y ^N^	
**NHS Wales [28]**	8	4	200	Y	350–750 ^S^	Y	Y	Y		Y	Y							Y	Y	Y		Y
**NHS Scotland [27]**	6	3	200	Y	200–600		Y				Y							“If risk factors”		Y		
**GP CPD Red Whale [30]**	4	1.5	100	Y		Y	Y	Y	Y		Y	Y			Y	Y		Y		Y	N ^F^	Y
**GIC guidance**																						
**London Transgender clinic (private)**	8	3	200	Y	400–700	Y	Y	Y	Y		Y							Y	Y	Y	N ^F^	
**Laurels NHS**	12	4	150	Y	200–600	Y	Y	Y	Y		Y							Y	Y	Y	N ^F^	
**Gender GP (private)**	6	2	200	Y	300–800	Y	Y	Y	Y			Y			Y			Y		Y ^P^		
**Sheffield NHS**	6	3	200	Y	300–600	Y	Y	Y		Y	Y				Y	Y		Y		Y	N ^F^	Y
**Tavistock NHS**	8	5	200	Y	400–600	Y	Y				Y							Y	Y	Y	N ^F^	Y ^Q^
**Nottingham NHS**	8	6	400	Y	400–600		Y	Y			Y		Y	Y						Y ^Q^		Y ^Q^
**Gendercare (private)**	10		100	Y	400–600 ^R^	Y	Y				Y							Y	Y	Y	Y	
**Leeds NHS**	8	6	400	Y	350–750 if aged < 40 yrs 300–600 if aged 40–50 yrs 200–400 if aged > 50 yrs or significant CV risk factors		Y	Y		Y	Y							Y		Y		

Key: Empty cell: no specific advice given; HBA1c, Glycated Haemoglobin; FSH&LH, Follicle Stimulating Hormone and Luteinizing hormone; SHBG, Sex hormone binding globulin; TSH, Thyroid Stimulating Hormone; ^A^,”strong recommendations”; ^B^, “level for premenopausal females (100 to 200 pg/mL)”; ^D^, If on spironolactone; ^E^, Consider screening at baseline. In individuals at low risk, screen at 60 years of age or if non compliant with hormone therapy; ^F^, Unless prolonged periods without sex hormones or additional risk factors; ^G^, Every 6 months; ^H^, Every 3 months; ^J^, Annually to document recovery after being on puberty suppression as required; ^K^, Yearly or as required; ^L^ consider to “fine tune hormone regimes”; ^M^, Dependent on age and length of exposure to estradiol; ^N^, from age 65 years on (earlier if risk factors); ^P^, advises if transwomen continue estradiol over 70 years of age they should “continue” receiving breast screening, although screening for younger transwomen is not specified; ^Q^, screening as per https://www.gov.uk/government/publications/nhs-population-screening-information-for-transgender-people/nhs-population-screening-information-for-trans-people (accessed on 1 Jan 2022); ^R^, consider stopping hormones at menopause age; ^S^, older patients that wish to reduce their dose may do so, with a target level of 200-300 pmol/L; CV, cardiovascular.

**Table 3:** Adverse childhood experiences, lifetime history of mental health problems and use of mental health services for non-gender related issues.

Problem	Number	%
*Adverse childhood experiences*		
Documented history of childhood abuse, neglect or violence (including “severe bullying” at school, n = 2)	13	19
*Lifetime history of mental health issue found in notes*		
Anxiety/Depression (mild/moderate/severe)	51	76
Personality Disorder	7	10
Deliberate Self Harm	36	54
Autistic Spectrum Disorder and/or Asperger’s Syndrome	10	15
Eating Disorder	2	3
Functional Seizures	3	4
Attention Deficit Hyperactivity Disorder	4	6
Obsessive Compulsive Disorder	3	4
Bipolar Type II	1	1
None of the above diagnoses	9	13
*Use of mental health services*		
Child and adolescent mental health service (CAMHS) or child psychiatry involvement for non-gender issues	24	36
Secondary psychiatric services’ involvement for non-gender issues (including referrals, assessments or admissions)	20	30
**Total number of patients**	67	100

## Data Availability

Data available on request due to privacy restrictions. The data presented in this study are available on request from the corresponding author. The data are not publicly available due to patient confidentiality.

## Supplementary Materials

The following supporting information can be downloaded at: https://www.mdpi.com/article/10.3390/healthcare10010121/s1, Table S1: Data collection spreadsheet.
